# Necrology

**Published:** 1897-12

**Authors:** 


### NECROLOGY.

### A. L. HUMMEL, M.D.

At Denver, Colorado, on October 24th, Dr. A. L. Hummel passed away after a serious illness of but ten days. He had resided in Colorado for the past three years as a more congenial climate for his impaired health, which up to the time of his last illness seemed much benefited by the change. But forty years of age, he had led a very active career. A graduate of the Medical Department of the University of Pennsylvania, he was the promoter of the University of Pennsylvania Press, and for several years the publisher of the JOURNAL OF COMPARATIVE MEDICINE AND VETERINARY ARCHIVES. Several other medical journals had been under his direction from time to time, and during the past five years he represented a syndicate of medical journal advertisers. He was buried at his old home, Hummelstown, Pa.

### JOHN H. ADAMSON, M.D.C.

At St. Paul, Minn., on October 25, 1897, Dr. John H. Adamson passed away, from injuries received by being kicked by an equine patient under his treatment, producing a double perforation of the small intestine, which surgical skill could not relieve.

Dr. Adamson was a graduate of the Chicago Veterinary College, class of 1893. His first year's studies were pursued in the Veterinary College, Edinburgh, Scotland. He came of a family of veterinarians, his grandfather, father, and brother were all practitioners of veterinary science in the old country.

He had filled for several years the chair of Anatomy in the United States College of Veterinary Surgeons. Dr. Adamson was a man of excellent habits and an able practitioner. He was thirty-nine years old, and leaves a wife to mourn his loss.

### WILLIAM HEYSER HARBAUGH, V.S.

In the death of Dr. Harbaugh the veterinary profession in Virginia has met an irreparable loss. A man of many parts, highly educated and an enthusiast in his chosen work, he had for years stood at the head of the profession in his adopted State. As a practitioner, he was favorably known throughout the State. As an author, his writings are known by all members of the profession throughout the country.

Dr. Harbaugh was born in Cumberland, Md., February 19, 1856, descending from the early settlers of that part of Frederick county known as Harbaugh's valley. He was educated at St. Vincent's College in Pennsylvania, and in Canada. He graduated as a veterinarian from the Toronto Veterinary College, Class of 1885, with high honors, winning the first prize on disease and treatment, first prize in microscopy, and the highest honors in entozoa, besides several second prizes in other branches.

In 1885 he removed to Richmond, Va., to begin the practice of his profession. He was the first graduated veterinarian in the State, and at once began a relentless warfare on the empiricism so prevalent throughout the South. His success as a practitioner was immediate, and he was at once recognized as an authority on all subjects pertaining to his profession throughout the State. His work as an inspector for the Bureau of Animal Industry and his writings on various subjects for that department are well known.

Perhaps his most important work was known best to his immediate associates. For years he had been impressed with the dangers of tuberculosis and its transmission to the human race from tuberculous cows. His work, against overwhelming odds, in trying to secure a better milk-supply for the city of Richmond rises to the plane of the highest philanthropy. In the face of a united opposition from the dairymen, and unaided by anyone, he kept up a relentless war upon tuberculous cows.

While tuberculosis is known to have existed in his family, he having lost a sister from the disease, those of us who knew him best feel certain that his life was sacrificed on the altar of scientific investigation. For weeks at a time his office and hospital was a place for practically demonstrating to medical men and laymen the existence of this dread disease among the dairy cows of Rich mond, and many think, and with good reason, that the continued work over diseased lungs and with the microscope resulted in a fatal infection.

Dr. Harbaugh was the first President of the Virginia State Veterinary Medical Association, and for the two terms during which he was President did more than anyone else for the advancement of its interests. He was more instrumental than any other one man in securing the necessary law for the protection of the veterinary practice of the State, and was an honored member of the State Board of Veterinary Medical Examiners.

Dr. Harbaugh leaves a wife and interesting family of small children, two girls and a boy, to mourn his loss. The profession at large mourns an earnest, conscientious worker, and the profession of this State a leader whose example may serve to stimulate a renewed devotion to their chosen work. His loss is felt by us all. Generous to a fault, to know him was to admire and respect. We mourn his loss. But we have with us his good example, which will always live.

GEO. C. FAVILLE,
Norfolk, Va.

